# Supplementary material for: HIVprotI: an integrated web based platform for prediction and design of HIV proteins inhibitors
Source: J Cheminform. 2018 Mar 9;10:12. doi: 10.1186/s13321-018-0266-y (PMC5845081; doi:10.1186/s13321-018-0266-y)
Supplement: Supplementary file 2 — Additional file 2. Source code of HIVProtI web server. [file 13321_2018_266_MOESM2_ESM.zip › HIVprotI_Source-code/hivprotI/weka-3-6-6/doc/weka/associations/gsp/Element.html]

Element


---


|  |  |  |
| --- | --- | --- |
| **Overview** \| **Package** \| **Class** \| **Tree** \| **Deprecated** \| **Index** \| **Help** \| **Weka's home** | |  |
| PREV CLASS   **NEXT CLASS** | **FRAMES**    **NO FRAMES**     **All Classes** |  |
| SUMMARY: NESTED \| FIELD \| CONSTR \| METHOD | DETAIL: FIELD \| CONSTR \| METHOD |  |


---


## weka.associations.gsp Class Element

```
java.lang.Object
  weka.associations.gsp.Element
```

**All Implemented Interfaces:**: java.io.Serializable, java.lang.Cloneable, RevisionHandler

---

``` public class Element extends java.lang.Object implements java.lang.Cloneable, java.io.Serializable, RevisionHandler ```

Class representing an Element, i.e., a set of events/items.

**Version:**
:   $Revision: 1.2 $

**Author:**
:   Sebastian Beer

**See Also:**: Serialized Form

---

**Constructor Summary**

`Element()`
  
          Constructor

`Element(int size)`
  
          Constructor accepting an initial size of the events Array as parameter.


| **Method Summary** | |
| --- | --- |
| `Element` | `clone()`             Returns a deep clone of an Element. |
| `boolean` | `containsOverOneEvent()`             Checks if an Element contains over one event. |
| `void` | `deleteEvent(java.lang.String position)`             Deletes the first or last event of an Element. |
| `boolean` | `equals(java.lang.Object obj)`             Checks if two Elements are equal. |
| `int[]` | `getEvents()`             Returns the events Array of an Element. |
| `static FastVector` | `getOneElements(Instances instances)`             Returns all events of the given data set as Elements containing a single event. |
| `java.lang.String` | `getRevision()`             Returns the revision string. |
| `boolean` | `isContainedBy(Instance instance)`             Checks if an Element is contained by a given Instance. |
| `boolean` | `isEmpty()`             Checks if the Element contains any events. |
| `static Element` | `merge(Element element1, Element element2)`             Merges two Elements into one. |
| `java.lang.String` | `toNominalString(Instances dataSet)`             Returns a String representation of an Element where the numeric value of each event/item is represented by its respective nominal value. |
| `java.lang.String` | `toString()`             Returns a String representation of an Element. |

**Methods inherited from class java.lang.Object**

`getClass, hashCode, notify, notifyAll, wait, wait, wait`

**Constructor Detail**

### Element

```
public Element()
```

:   Constructor

---


### Element

```
public Element(int size)
```

:   Constructor accepting an initial size of the events Array as parameter.

    **Parameters:**: `size` - the size


**Method Detail**

### getOneElements

```
public static FastVector getOneElements(Instances instances)
```

:   Returns all events of the given data set as Elements containing a single
    event. The order of events is determined by the header information of
    the corresponding ARFF file.

    :   **Parameters:**: `instances` - the data set **Returns:**: the set of 1-Elements

---


### merge

```
public static Element merge(Element element1,
                            Element element2)
```

:   Merges two Elements into one.

    :   **Parameters:**: `element1` - first Element: `element2` - second Element **Returns:**: the merged Element

---


### clone

```
public Element clone()
```

:   Returns a deep clone of an Element.

    :   **Overrides:**: `clone` in class `java.lang.Object`
    :   **Returns:**: the cloned Element

---


### containsOverOneEvent

```
public boolean containsOverOneEvent()
```

:   Checks if an Element contains over one event.

    :   **Returns:**: true, if the Element contains over one event, else false

---


### deleteEvent

```
public void deleteEvent(java.lang.String position)
```

:   Deletes the first or last event of an Element.

    :   **Parameters:**: `position` - the position of the event to be deleted (first or last)

---


### equals

```
public boolean equals(java.lang.Object obj)
```

:   Checks if two Elements are equal.

    :   **Overrides:**: `equals` in class `java.lang.Object`
    :   **Returns:**: true, if the two Elements are equal, else false

---


### getEvents

```
public int[] getEvents()
```

:   Returns the events Array of an Element.

    :   **Returns:**: the events Array

---


### isContainedBy

```
public boolean isContainedBy(Instance instance)
```

:   Checks if an Element is contained by a given Instance.

    :   **Parameters:**: `instance` - the given Instance **Returns:**: true, if the Instance contains the Element, else false

---


### isEmpty

```
public boolean isEmpty()
```

:   Checks if the Element contains any events.

    :   **Returns:**: true, if the Element contains no event, else false

---


### toNominalString

```
public java.lang.String toNominalString(Instances dataSet)
```

:   Returns a String representation of an Element where the numeric value
    of each event/item is represented by its respective nominal value.

    :   **Parameters:**: `dataSet` - the corresponding data set containing the header information **Returns:**: the String representation

---


### toString

```
public java.lang.String toString()
```

:   Returns a String representation of an Element.

    :   **Overrides:**: `toString` in class `java.lang.Object`
    :   **Returns:**: the String representation

---


### getRevision

```
public java.lang.String getRevision()
```

:   Returns the revision string.

    :   **Specified by:**: `getRevision` in interface `RevisionHandler`
    :   **Returns:**: the revision


---


|  |  |  |
| --- | --- | --- |
| **Overview** \| **Package** \| **Class** \| **Tree** \| **Deprecated** \| **Index** \| **Help** \| **Weka's home** | |  |
| PREV CLASS   **NEXT CLASS** | **FRAMES**    **NO FRAMES**     **All Classes** |  |
| SUMMARY: NESTED \| FIELD \| CONSTR \| METHOD | DETAIL: FIELD \| CONSTR \| METHOD |  |


---
